# Supplementary material for: Dynamic interactions between E-cadherin and Ankyrin-G mediate epithelial cell polarity maintenance
Source: Nat Commun. 2023 Oct 27;14:6860. doi: 10.1038/s41467-023-42628-1 (PMC10611751; doi:10.1038/s41467-023-42628-1)
Supplement: Supplementary file 1 — Supplementary Information [file 41467_2023_42628_MOESM1_ESM.pdf]

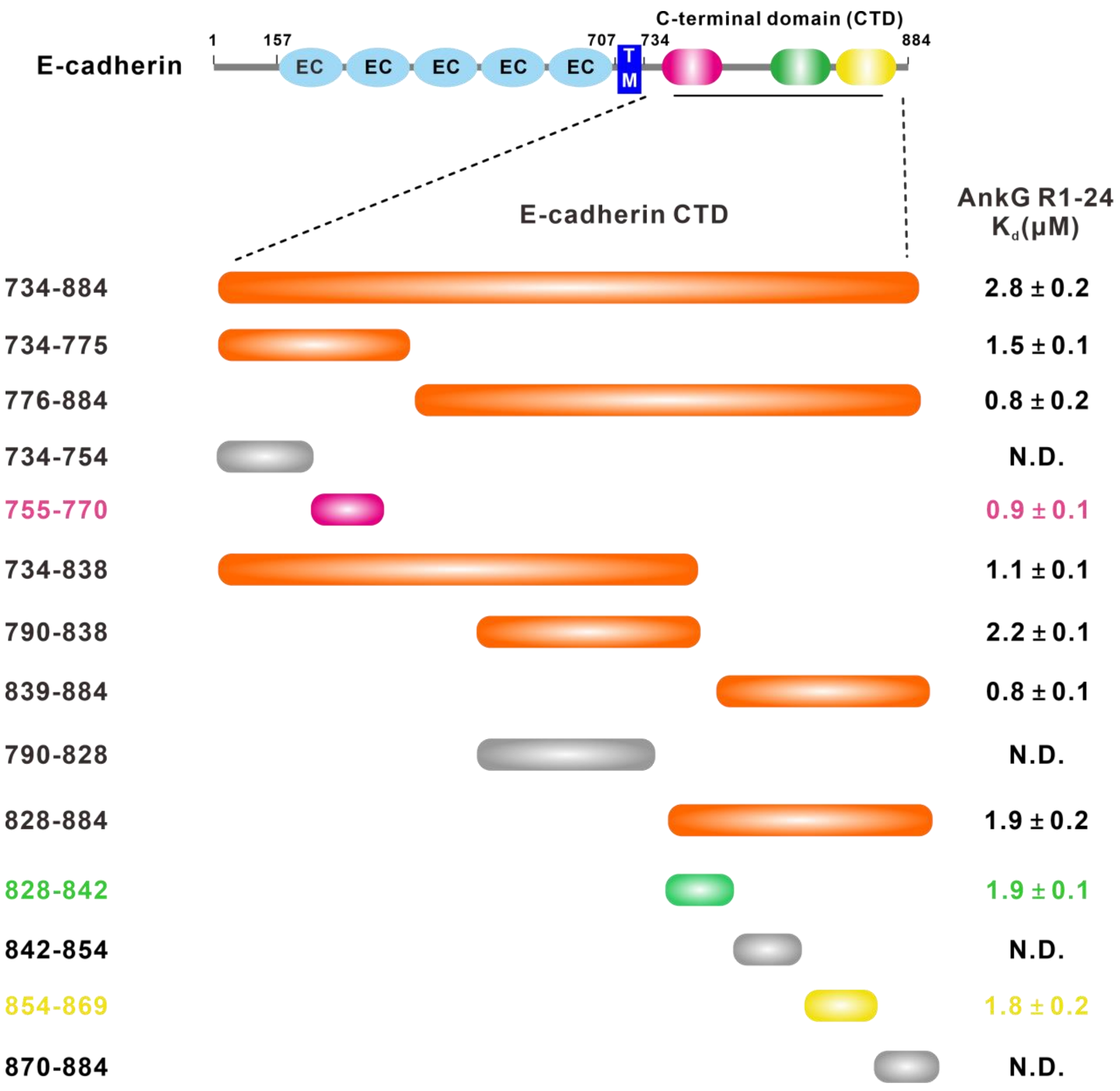

Supplementary Fig. 1 ITC-based mapping of the E-cadherin CTD region in binding with AnkG.

ITC-based mapping of the minimal AnkG binding region in the E-cadherin. N.D. indicates that no binding was detected.

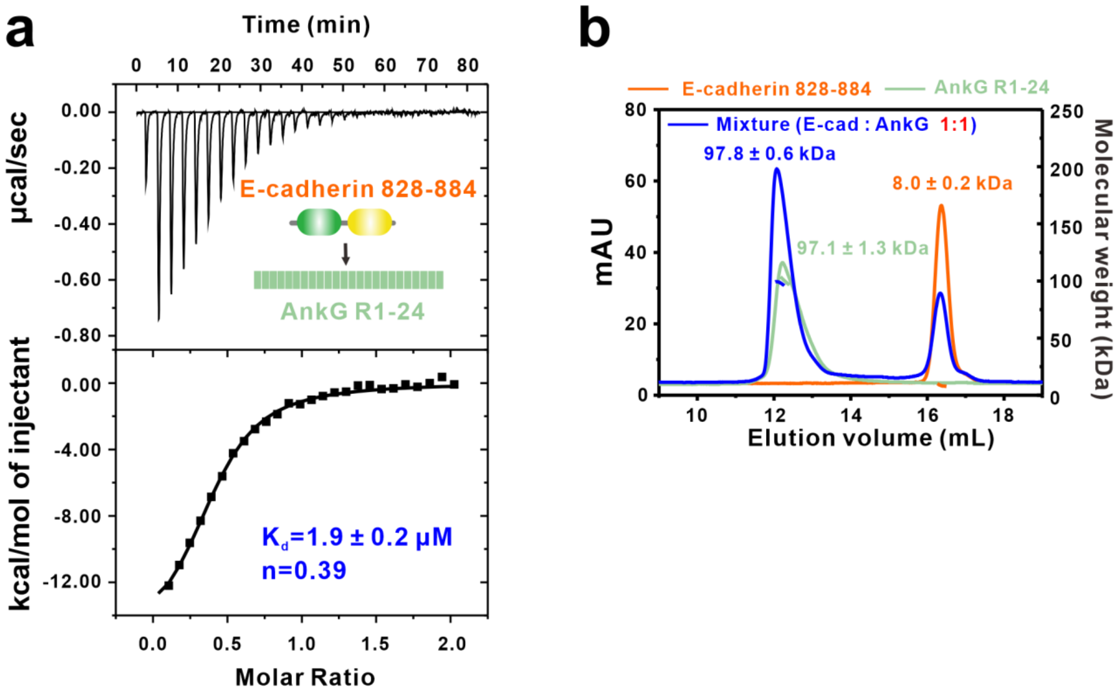

Supplementary Fig. 2 The binding stoichiometry for E-cadherin 828-884 and AnkG complex was 1:1.

(a) ITC-based measurement of the binding affinity of E-cadherin 828-884 with AnkG R1-24. **(b)** Analytical gel filtration chromatography coupled with static light scattering analysis of E-cadherin 828-884 (orange), AnkG R1-24 (green), and the E-cadherin 828-884−AnkG R1-24 complex (blue) at a 1:1 molar ratio.

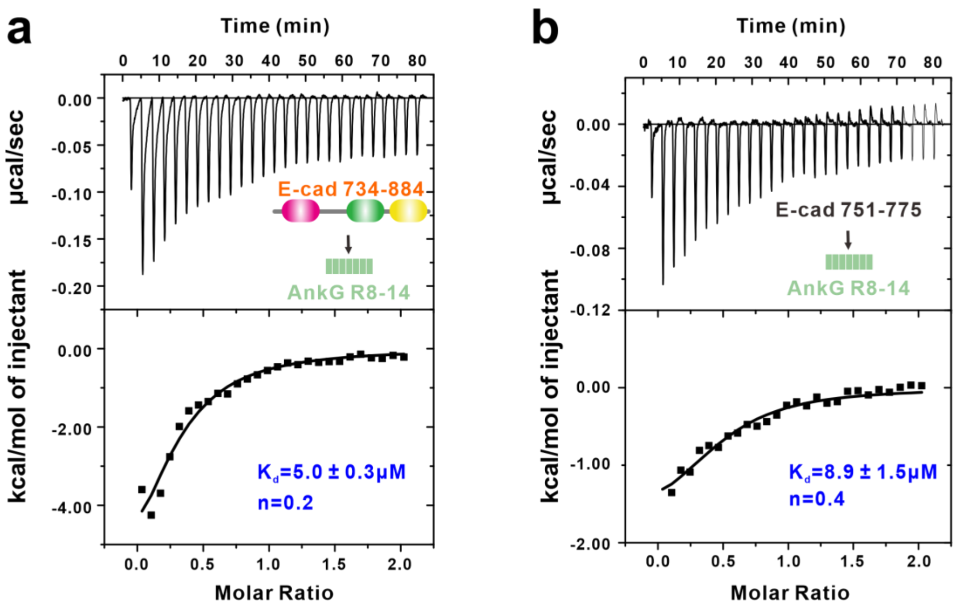

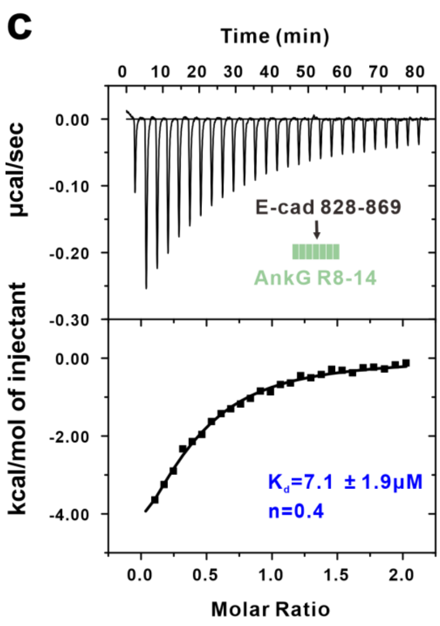

d

E-cadherin	AnkG	K_d (µM)	n value
751-775	R6-24	1.8 ± 0.2	0.47
751-775	R8-24	1.3 ± 0.3	0.47
751-775	R8-14	8.9 ± 1.5	0.40
828-869	R6-24	1.0 ± 0.1	0.45
828-869	R8-24	2.5 ± 0.3	0.86
828-869	R8-14	7.1 ± 1.9	0.40
734-884	R6-24	2.9 ± 0.3	0.21
734-884	R8-24	1.5 ± 0.1	0.18

Supplementary Fig. 3 ITC-based mapping of the minimal E-cadherin binding region on AnkG.

(a-c) ITC-based measurements of the binding affinities of AnkG R8-14 with E-cadherin residues 734-884 (CTD) (**a**), E-cadherin residues 751-775 (site-1) (**b**), and E-cadherin 828-869 (including site-2 and site-3) (**c**). **(d)** ITC-based measurement of binding affinities between AnkG fragments (R6-24, R8-24, and R8-14) and E-cadherin CTD fragments (residues 751-775, residues 828-869, and residues 734-884).

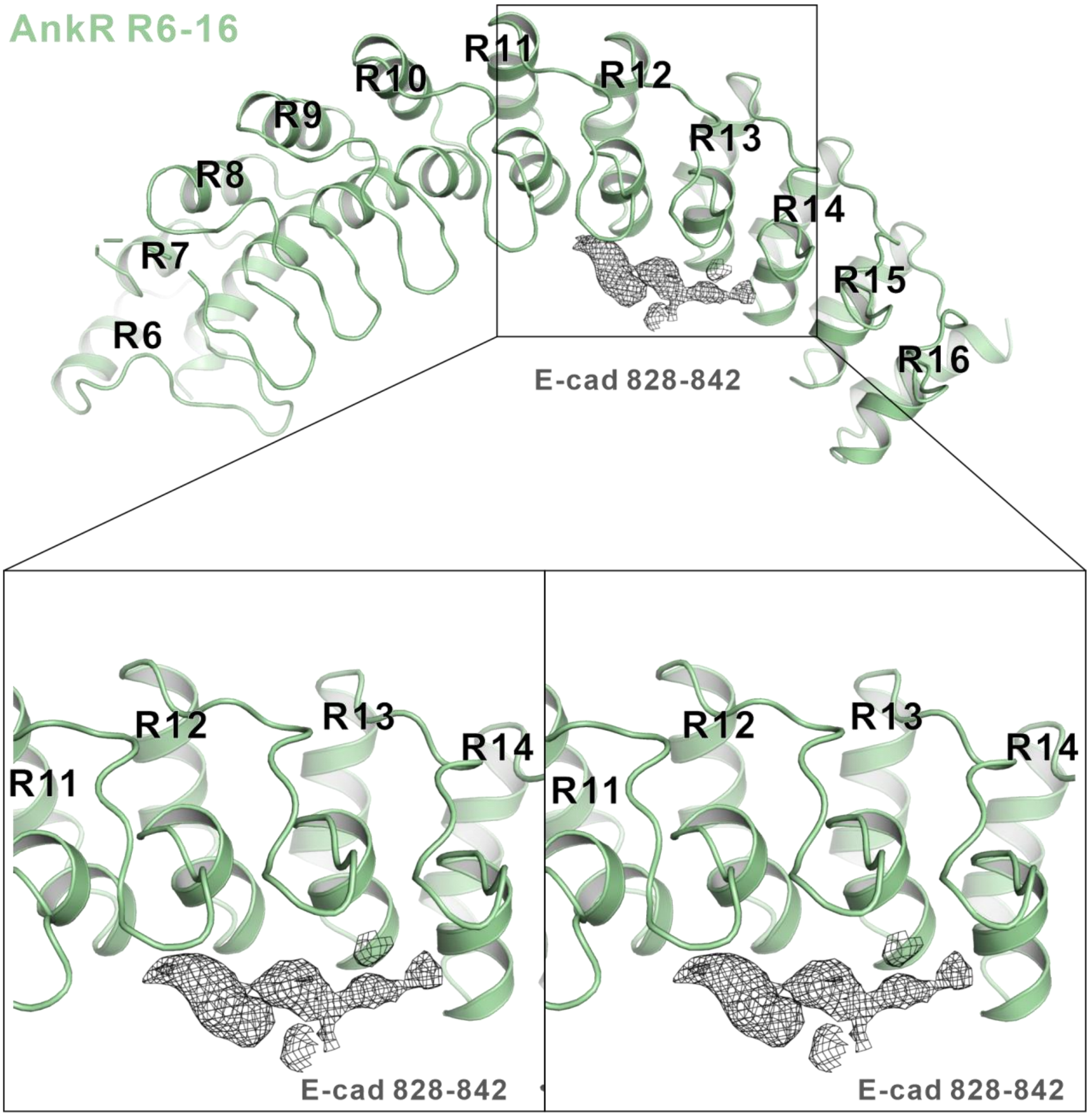

Supplementary Fig. 4 The crystal structure of AnkR R6-16 in a complex with E-cadherin 828-842 (site-2).

The Fo-Fc map was contoured at 2.5 σ.

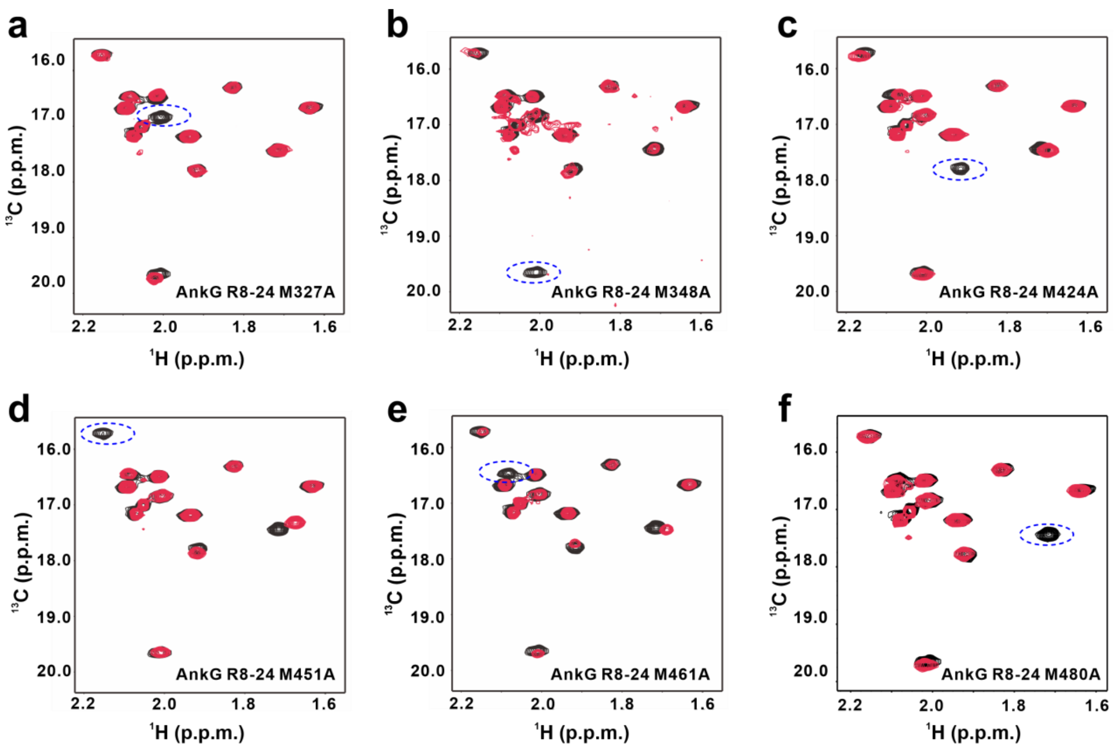

Supplementary Fig. 5 [^{1}H–^{13}C]-correlated methyl-TROSY spectra of Met-$^{13}CH_3$ isotopically labeled AnkG R8-24.

(a-f) The spectra of individual variants, M327A **(a)**, M348A **(b)**, M424A **(c)**, M451A **(d)**, M461A **(e)**, and M480A **(f)**, were overlaid with WT spectrum (black) to achieve the Met assignments.

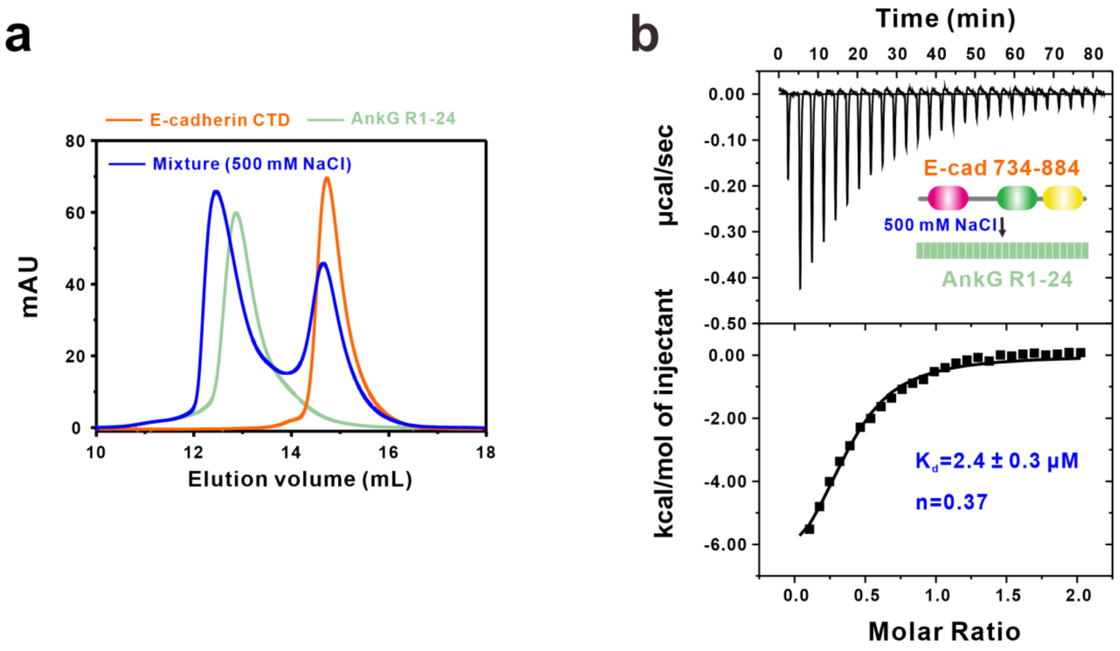

Supplementary Fig. 6 Charge–charge interactions merely contribute to the binding between E-cadherin and AnkG.

(a) Analytical gel filtration chromatography of E-cadherin CTD (orange), AnkG R1-24 (green), and E-cadherin CTD−AnkG R1-24 complex (blue) at a 1:1 molar ratio with the buffer containing 50 mM Tris, 500 mM NaCl, 1 mM DTT and 1 mM EDTA at pH 7.8. **(b)** ITC-based measurement of the binding affinity between the E-cadherin CTD and AnkG R1-24 with the same buffer.

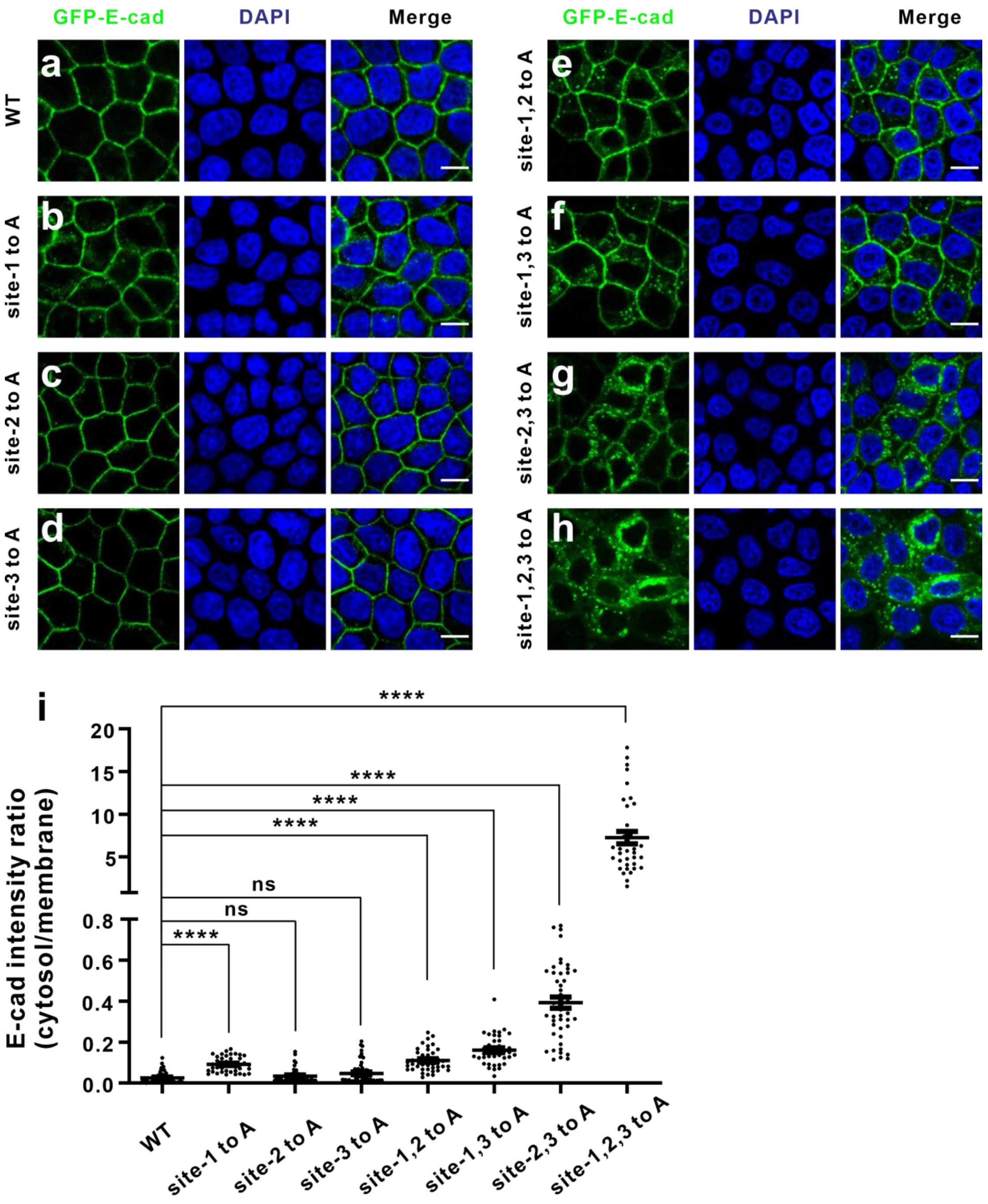

Supplementary Fig. 7 AnkG stabilized the localization of E-cadherin on the lateral membranes.

(a-h) MDCK cells were transfected with wild-type (WT) GFP-E-cadherin (**a**), GFP-E-cadherin site-1 to A (**b**), GFP-E-cadherin site-2 to A (**c**), GFP-E-cadherin site-3 to A (**d**), GFP-E-cadherin site-1, 2 to A (**e**), GFP-E-cadherin site-1, 3 to A (**f**), GFP-E-cadherin site-2, 3 to A (**g**), and GFP-E-cadherin site-1, 2, 3 to A (**h**). The cells were stained with GFP signals showing E-cadherin localization. Scale bars: 10 μm. **(i)** Quantification of the immunofluorescence intensity ratios of cytosolic to membranes

for E-cadherin WT (n=66); site-1 to A (n=41); site-2 to A (n=54); site-3 to A (n=62); site-1, 2 to A (n=40); site-1, 3 to A (n=39); site-2, 3 to A (n=45); and site-1, 2, 3 to A (n=36). Data are presented as the means ± SEMs and analyzed using one way ANOVA followed by Dunnett's multiple comparisons test to WT E-cadherin, ****$p<0.0001$, ns, not significant.

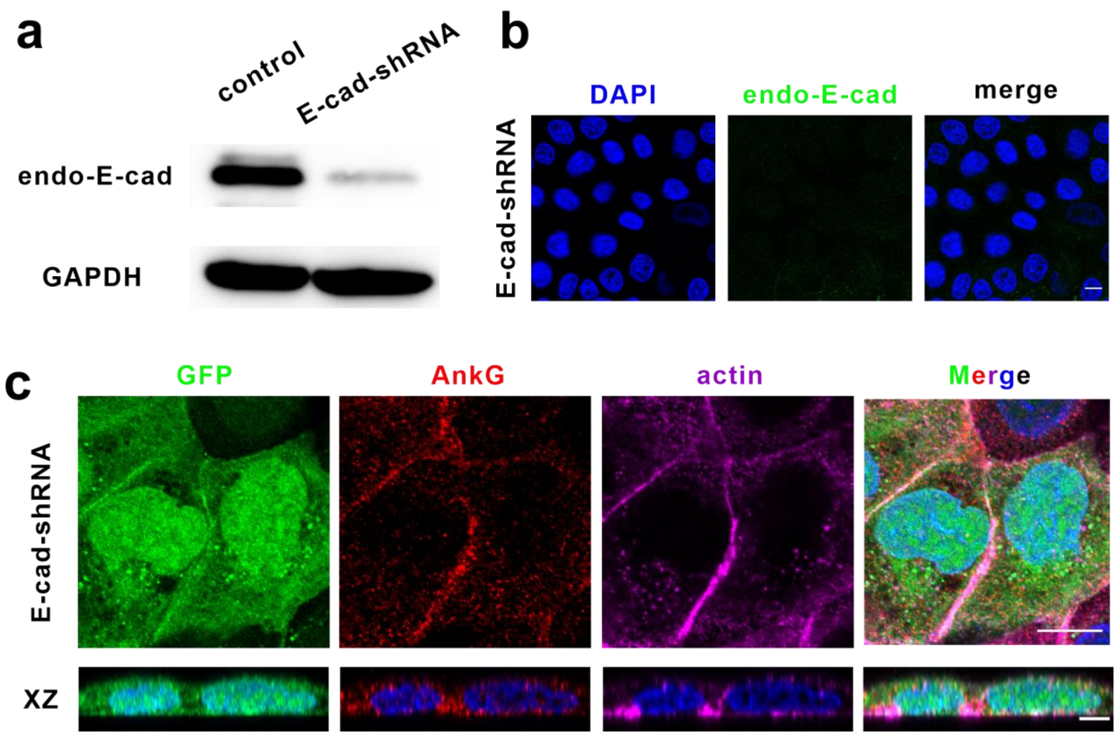

Supplementary Fig. 8 Generation of the stable MDCK cell line expressing a shRNA targeting E-cadherin.

(a) Western blot of endogenous E-cadherin in either control shRNA- (left) or E-cadherin-shRNA-transfected MDCK cells (right). GAPDH was used as a loading control. **(b)** Representative fluorescence images of MDCK cells stably expressing a shRNA targeting E-cadherin. Scale bars: 10 μm. **(c)** MDCK cells stably expressing a shRNA targeting E-cadherin were transfected with the GFP vector as a control and stained with AnkG and actin signals (related to Figure 5). Scale bars: 10 μm. XZ projection is shown at bottom. Scale bars: 5 μm.

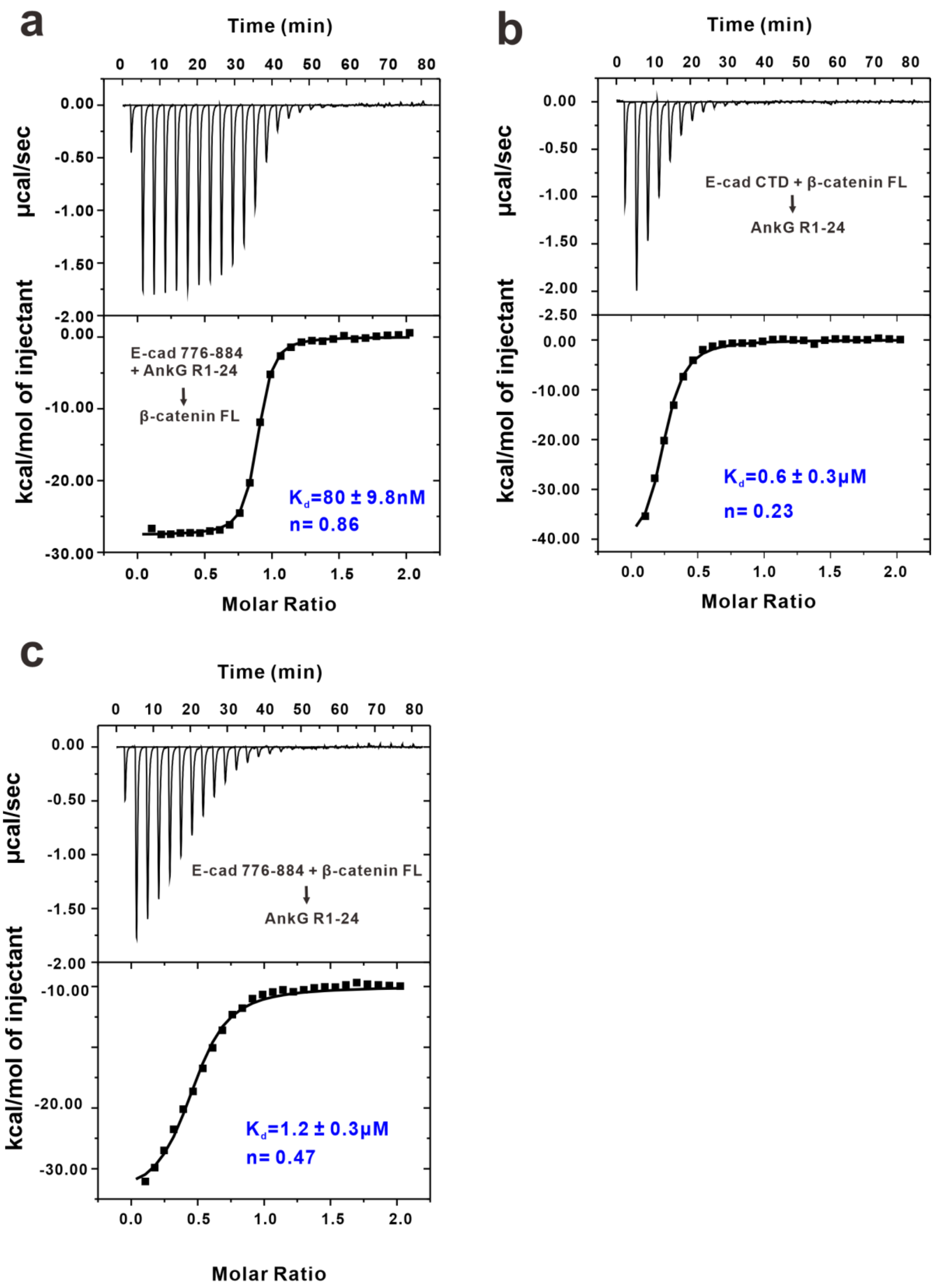

Supplementary Fig. 9 ITC data confirmed the formation of a three-subunit complex.

(a) ITC-based measurement of the binding affinity of the E-cadherin 776-884−AnkG R1-24 complex with β-catenin FL (full length). (**b**) ITC-based measurement of the binding affinity of the E-cadherin CTD−β-catenin complex with AnkG R1-24. (**c**) ITC-based measurement of the binding affinity of the E-cadherin 776-884−β-catenin complex with AnkG R1-24.

Supplementary Table 1 Statistics of X-ray crystallographic data collection and model refinement

**Data collection**	
Data sets	AnkR/E-cadherin 828-842
Space group	$P2_12_12_1$
Wavelength (Å)	0.97918
Unit Cell Parameters (Å)	a=82.985, b=82.976
	c=152.969
	α=β=γ=90°
Resolution range (Å)	50-3.00 (3.05-3.00)
No. of unique reflections	22175 (1088)
Redundancy	7.3 (7.8)
I/σ	13.1 (1.0)
Completeness (%)	99.9 (100)
R_{merge} [a] (%)	12.3 (103.8)
$CC_{1/2}$ [b]	0.985 (0.666)
**Structure refinement**	
Resolution (Å)	50-3.00 (3.09-3.00)
R_{cryst} [c]/R_{free} [d] (%)	22.71/25.12 (31.33/30.64)
rmsd bonds (Å)/angles (°)	0.007/1.269
Average B factor ($Å^2$) [e]	98.5
No. of atoms	
Protein atoms	5045
Water	3
Ligands	5
No. of reflections	
Working set	21029 (1096)
Test set	2030 (107)
Ramachandran plot regions [d]	
Favored (%)	93.96
Allowed (%)	6.04
Outliers (%)	0

Numbers in parentheses represent the value for the highest resolution shell.

a. $R_{merge} = \Sigma |I_i - \langle I \rangle| / \Sigma I_i$, where I_i is the intensity of the measured reflection and $\langle I \rangle$ is the mean intensity of all symmetry-related reflections.

b. $CC_{1/2}$ were defined by Karplus and Diederichs [*1*].

c. $R_{cryst}=\Sigma||F_{calc}| - |F_{obs}||/\Sigma F_{obs}$, where F_{obs} and F_{calc} are the observed and calculated structure factors.

d. $R_{free}= \Sigma_T||F_{calc}| - |F_{obs}||/\Sigma F_{obs}$, where T is a test data set of approximately 5% of the total unique reflections randomly chosen and set aside prior to refinement.

e. B factors and Ramachandran plot statistics are calculated using MOLPROBITY [*2*].

Supplementary Table 2 List of primer sequences used in this study

Primer name	Primer sequences (5'-3')
E-cadherin-734-BamHI up	ggatcccggaggagaacggtggtc
E-cadherin-884-XhoI dn	ctcgagctagtcgtcctcaccaccgcc
E-cadherin-755-BamHI up	ggatcctattactatgatgaagaag
E-cadherin-770-XhoI dn	ctcgagctaatcaaagtcctggtcttc
E-cadherin-828-BamHI up	ggatccccttacgactctctgttg
E-cadherin-842-XhoI dn	ctcgagctaagaaccactgccctcgta
E-cadherin-854-BamHI up	ggatccccttacgactctctgttg
E-cadherin-869-XhoI dn	ctcgagctagttgccccactcgttcag
E-cadherin-776-BamHI up	ggatccaggggcctggatgcccga
E-cadherin-775-XhoI dn	ctcgagctagtgcagctggctcaaatc
E-cadherin-790-BamHI up	ggatccgctcccaccctcatgagc
E-cadherin-838-XhoI dn	ctcgagctactcgtaatcgaacaccaa
E-cadherin-839-BamHI up	ggatccggcagtggttctgaagcc
E-cadherin-828-XhoI dn	ctcgagctaagggggtgccgtggggtc
E-cadherin-854-XhoI dn	ctcgagctactcagaggagttcagtga
E-cadherin-870-BamHI up	ggatcccgattcaagaagctggcg
E-cadherin-Y753A-up	acccgggacaacgttgcttactatgatgaagaa
E-cadherin-Y753A-dn	ttcttcatcatagtaagcaacgttgtcccgggt
E-cadherin-Y754A-up	cgggacaacgtttatgcctatgatgaagaagga
E-cadherin-Y754A-dn	tccttcttcatcataggcataaacgttgtcccg
E-cadherin-Y755A-up	gacaacgtttattacgctgatgaagaaggaggc
E-cadherin-Y755A-dn	gcctccttcttcatcagcgtaataaacgttgtc
E-cadherin-Y827A-up	cccacagccccgcctgctgattctctgctcgtg
E-cadherin-Y827A-dn	cacgagcagagaatcagcaggcggggctgtggg
E-cadherin-Y835A-up	ctgctcgtgtttgacgctgaaggaagcggttcc
E-cadherin-Y835A-dn	ggaaccgcttccttcagcgtcaaacacgagcag
E-cadherin-Y859A-up	gacaaagaccaggacgctgactacttgaacgaa
E-cadherin-Y859A-dn	ttcgttcaagtagtcagcgtcctggtctttgtc
E-cadherin-Y861A-up	gaccaggactatgacgccttgaacgaatggggc
E-cadherin-Y861A-dn	gccccattcgttcaaggcgtcatagtcctggtc
E-cadherin-W865A-up	gactacttgaacgaagcgggcaatcgcttcaag
E-cadherin-W865A-dn	cttgaagcgattgcccgcttcgttcaagtagtc
AnkG-38-EcoRI-up	gaattcaagtcggatgccaatgca
AnkG-855-SalI-dn	gtcgacctatgacatgtcgaggacttc
AnkG-M327A-up	caggtggtagaggcgttgctggacaga
AnkG-M327A-dn	tctgtccagcaacgcctctaccacctg
AnkG-M348A-up	tccccactgcacgcggccacacaagga
AnkG-M348A-dn	tccttgtgtggccgcgtgcagtgggga
AnkG-M424A-up	cgaatccgagtagcggaactccttttg
AnkG-M424A-dn	caaaaggagttccgctactcggattcg

AnkG-M451A-up	gtcgctgccttcgcgggacatgtaaat
AnkG-M451A-dn	atttacatgtcccgcgaaggcagcgac
AnkG-M461A-up	gtgtcacagctagcgcatcatggagcc
AnkG-M461A-dn	ggctccatgatgcgctagctgtgacac
AnkG-M480A-up	acagcattgcatgcggcggctcggtcg
AnkG-M480A-dn	cgaccgagccgccgcatgcaatgctgt
AnkG-275-EcoRI-up	gaattcaatgacatcactcctttg
AnkG-505-SalI-dn	gtcgacctacttagcttttgcttctac
AnkG-198-EcoRI-up	gaattcaagggaaaagtgcgcctc
β-catenin-1-BamHI-up	ggatccatggctactcaagctgacctgatg
β-catenin-781-XhoI-dn	ctcgagttacaggtcagtatcaaaccaggccag
p120 catenin-1-EcoRI-up	gaattcatggacgactcagaggtggagtcg
p120 catenin-938-NotI-dn	gcggccgcctaaatcttctgcatcaagggtgct
p120catenin-324-EcoRI-up	gaattcatgattggtgaagaggtgccgcct

References

1. Karplus, P.A. and K. Diederichs, *Linking crystallographic model and data quality.* Science, 2012. **336**(6084): p. 1030-3.
2. Chen, V.B., et al., *MolProbity: all-atom structure validation for macromolecular crystallography.* Acta Crystallographica. Section D, Biological Crystallography, 2010. **66**: p. 12-21.
