# Supplementary material for: Dynamic interactions between E-cadherin and Ankyrin-G mediate epithelial cell polarity maintenance
Source: Nat Commun. 2023 Oct 27;14:6860. doi: 10.1038/s41467-023-42628-1 (PMC10611751; doi:10.1038/s41467-023-42628-1)
Supplement: Supplementary file 3 — Reporting Summary [file 41467_2023_42628_MOESM3_ESM.pdf]

nature portfolio

Corresponding author(s): Chao Wang; Chengdong Huang

Last updated by author(s): Oct 7, 2023

Reporting Summary

Nature Portfolio wishes to improve the reproducibility of the work that we publish. This form provides structure for consistency and transparency in reporting. For further information on Nature Portfolio policies, see our Editorial Policies and the Editorial Policy Checklist.

Statistics

For all statistical analyses, confirm that the following items are present in the figure legend, table legend, main text, or Methods section.

n/a	Confirmed	
☐	☒	The exact sample size (*n*) for each experimental group/condition, given as a discrete number and unit of measurement
☒	☐	A statement on whether measurements were taken from distinct samples or whether the same sample was measured repeatedly
☐	☒	The statistical test(s) used AND whether they are one- or two-sided<br>*Only common tests should be described solely by name; describe more complex techniques in the Methods section.*
☒	☐	A description of all covariates tested
☒	☐	A description of any assumptions or corrections, such as tests of normality and adjustment for multiple comparisons
☐	☒	A full description of the statistical parameters including central tendency (e.g. means) or other basic estimates (e.g. regression coefficient) AND variation (e.g. standard deviation) or associated estimates of uncertainty (e.g. confidence intervals)
☐	☒	For null hypothesis testing, the test statistic (e.g. *F*, *t*, *r*) with confidence intervals, effect sizes, degrees of freedom and *P* value noted<br>*Give P values as exact values whenever suitable.*
☒	☐	For Bayesian analysis, information on the choice of priors and Markov chain Monte Carlo settings
☒	☐	For hierarchical and complex designs, identification of the appropriate level for tests and full reporting of outcomes
☒	☐	Estimates of effect sizes (e.g. Cohen's *d*, Pearson's *r*), indicating how they were calculated

Our web collection on statistics for biologists contains articles on many of the points above.

Software and code

Policy information about availability of computer code

Data collection	All software names were indicated in Methods section. Zeiss LSM 880; NMRPipe program
Data analysis	All software names were indicated in Methods section. ImageJ; NMRViewJ software; PHASER software; COOT; PHENIX; PyMol; Graphpad

For manuscripts utilizing custom algorithms or software that are central to the research but not yet described in published literature, software must be made available to editors and reviewers. We strongly encourage code deposition in a community repository (e.g. GitHub). See the Nature Portfolio guidelines for submitting code & software for further information.

Data

Policy information about availability of data

All manuscripts must include a data availability statement. This statement should provide the following information, where applicable:

- Accession codes, unique identifiers, or web links for publicly available datasets
- A description of any restrictions on data availability
- For clinical datasets or third party data, please ensure that the statement adheres to our policy

All data needed to evaluate the conclusions in the paper are present in the paper and/or the Supplementary Materials. Data supporting the findings of this manuscript are available from the corresponding author upon reasonable request. A reporting summary for this article is available as a Supplementary Information file. The source data underlying Figs. 6i, 6j, 6l, Figs. 7s, 7t, 7u and Supplementary Figs. 7i and 8a are provided as a Source Data file.

Research involving human participants, their data, or biological material

Policy information about studies with human participants or human data. See also policy information about sex, gender (identity/presentation), and sexual orientation and race, ethnicity and racism.

Reporting on sex and gender	Not involved
Reporting on race, ethnicity, or other socially relevant groupings	Not involved
Population characteristics	Not involved
Recruitment	Not involved
Ethics oversight	Not involved

Note that full information on the approval of the study protocol must also be provided in the manuscript.

Field-specific reporting

Please select the one below that is the best fit for your research. If you are not sure, read the appropriate sections before making your selection.

☒ Life sciences ☐ Behavioural & social sciences ☐ Ecological, evolutionary & environmental sciences

For a reference copy of the document with all sections, see nature.com/documents/nr-reporting-summary-flat.pdf

Life sciences study design

All studies must disclose on these points even when the disclosure is negative.

Sample size	We counted the number of samples per group of cells and the n number is provided and defined as a precise value.
Data exclusions	No exclusion of data was performed in this study.
Replication	For cellular imaging and WB experiments, the experimental findings were dependably reproduced at least three times.
Randomization	Samples were groups according to the plasmids transfected (for cell lines). So, the randomization is not relevant to each group.
Blinding	For cellular data from Fig. 6 and Fig. 7, the blinded plasmids were used. When we transfected the virus of WT and various mutants of E-cadherin, one person labeled these virus with the cood of a, b, c and so on. The following experiments and quantifications were made under blindness. When all the experiments were completed, the corresponding codes were uncovered.

Reporting for specific materials, systems and methods

We require information from authors about some types of materials, experimental systems and methods used in many studies. Here, indicate whether each material, system or method listed is relevant to your study. If you are not sure if a list item applies to your research, read the appropriate section before selecting a response.

Materials & experimental systems

n/a	Involved in the study	
☐	☒	Antibodies
☐	☒	Eukaryotic cell lines
☒	☐	Palaeontology and archaeology
☒	☐	Animals and other organisms
☒	☐	Clinical data
☒	☐	Dual use research of concern
☒	☐	Plants

Methods

n/a	Involved in the study	
☒	☐	ChIP-seq
☒	☐	Flow cytometry
☒	☐	MRI-based neuroimaging

Antibodies

Antibodies used	Rabbit anti-E-cadherin (Proteintech 20874-1-AP; 1:1000 for western blotting (WB); 1:500 for immunofluorescence (IF)); anti-AnkG (4G3F8; Life Technologies, 1:500 for IF); Alexa Fluor 647-Phalloidin (Invitrogen, A22287, 1:1000 for IF); Rabbit anti-GAPDH (Proteintech, 10494-1-AP, 1:2000 for WB); Rabbit anti-p120 catenin (Proteintech, 12180-1-AP, 1:400 for IF) Rabbit anti-β-catenin (CST, 8480; 1:400 for IF), mouse anti-α-catenin (13-9700; 1:400 for IF) antibodies was from Thermo Fisher Scientific. Secondary goat

	antibodies conjugated to Alexa Fluor 488, 568, or 647 (Thermo Fisher) were used at 1:1000 dilutions.
Validation	We checked all the validation statements on the manufacturer's websites of these antibodies and our cellular data and we confirmed the validation of these antibodies.

Eukaryotic cell lines

Policy information about cell lines and Sex and Gender in Research

Cell line source(s)	HEK 293: ATCC ; MDCK: ATCC
Authentication	The cell line used in this study was not further authenticated.
Mycoplasma contamination	The cell lines were not tested for mycoplasma contamination.
Commonly misidentified lines (See ICLAC register)	The cell line used was not found to be on the list of commonly misidentified cell lines (International Cell Line Authentication Committee).
